# Phosphorus Concentration in Water Affects the Biofilm Community and the Produced Amount of Extracellular Polymeric Substances in Reverse Osmosis Membrane Systems

**DOI:** 10.3390/membranes11120928

**Published:** 2021-11-26

**Authors:** Luisa Javier, Laura Pulido-Beltran, Joop Kruithof, Johannes S. Vrouwenvelder, Nadia M. Farhat

**Affiliations:** 1 Water Desalination and Reuse Center (WDRC), Division of Biological and Environmental Science and Engineering (BESE), King Abdullah University of Science and Technology (KAUST), Thuwal 23955-6900, Saudi Arabia; luisa.javier@kaust.edu.sa (L.J.); laura.pulidobeltran@kaust.edu.sa (L.P.-B.); johannes.vrouwenvelder@kaust.edu.sa (J.S.V.); 2 Wetsus, European Centre of Excellence for Sustainable Water Technology, Oostergoweg 9, 8911 MA Leeuwarden, The Netherlands; joop.kruithof@planet.nl; 3 Faculty of Applied Sciences, Department of Biotechnology, Delft University of Technology, Van der Maasweg 9, 2629 HZ Delft, The Netherlands

**Keywords:** phosphate limitation, bacterial communities, P limitation, membrane cleaning, reverse osmosis seawater desalination

## Abstract

Biofouling is a problem that hinders sustainable membrane-based desalination and the stratification of bacterial populations over the biofilm’s height is suggested to compromise the efficiency of cleaning strategies. Some studies reported a base biofilm layer attached to the membrane that is harder to remove. Previous research suggested limiting the concentration of phosphorus in the feed water as a biofouling control strategy. However, the existence of bacterial communities growing under phosphorus-limiting conditions and communities remaining after cleaning is unknown. This study analyzes the bacterial communities developed in biofilms grown in membrane fouling simulators (MFSs) supplied with water with three dosed phosphorus conditions at a constant biodegradable carbon concentration. After biofilm development, biofilm was removed using forward flushing (an easy-to-implement and environmentally friendly method) by increasing the crossflow velocity for one hour. We demonstrate that small changes in phosphorus concentration in the feed water led to (i) different microbial compositions and (ii) different bacterial-cells-to-EPS ratios, while (iii) similar bacterial biofilm populations remained after forward flushing, suggesting a homogenous bacterial community composition along the biofilm height. This study represents an exciting advance towards greener desalination by applying non-expensive physical cleaning methods while manipulating feed water nutrient conditions to prolong membrane system performance and enhance membrane cleanability.

## 1. Introduction

In the Arab region, fourteen countries rely on non-conventional water treatment, such as desalination, to meet their growing water demand [1]. Reverse osmosis membrane systems have significantly expanded in the Arab region, where half of the world’s desalinated water is produced [2]. One of the main challenges in desalination is to produce clean water at a lower cost. The water cost can be impacted by the occurrence of biofouling. Biofouling occurs when biofilm, or the accumulation of bacteria embedded in a matrix of extracellular polymeric substances (EPS), excessively accumulates on the membrane and feed spacer, resulting in an unacceptable decline in membrane performance [3]. Biofouling is considered a problem in achieving sustainable desalination, as it impacts the membrane’s operational parameters, causing, for example, an increase in feed channel pressure drop, a decline in flux, and the passage of salt [4].

Nutrient conditions in the feed water alter the growth of different bacterial communities and substances segregated in the biofilms [5]. It was suggested that a mass ratio of carbon (C), nitrogen (N), and phosphorus (P) of at least ~100:23:4.3 is needed for bacterial growth to occur [6]. The demand for C, N, and P increases as the bacterial growth rate increases. C:N ratios appear to vary slightly in time even after the growth rate increases. However, a minor change in phosphorus concentration in the feed water impacts the growth of microorganisms [7,8,9]. One of the strategies proposed for biofouling control is manipulating the feed water nutrient composition [10]. The limitation of phosphorus in the feed water has been suggested as an approach for biofouling control [11,12,13]. Previously it was demonstrated that even at extremely low phosphorus concentrations in the feed water (≤0.3 µg PO_4_-P·L^−1^) at high assimilable organic carbon concentration, an adverse effect on the feed channel pressure drop was observed, as bacteria increased the production of EPS [9]. This finding suggests that certain bacterial families outcompete and adapt to phosphorus-limiting conditions.

Bacterial adaptations to phosphorus-limiting conditions include, but are not limited to, (i) increased EPS production, (ii) phosphorus accumulation, regeneration, and sequestration, and (iii) regulation of adhesin protein production. Danhorn et al. (2004) [14] and Desmond et al. (2018) [15] showed that phosphorus limitation enhances biofilm formation by bacteria increasing the production of EPS to provide bacterial protection from a wide range of stresses [16]. Similarly, some bacterial families adapt to phosphorus-limiting conditions by accumulating, regenerating, or sequestering phosphorus. Polyphosphate-accumulating organisms (PAO) accumulate phosphorus through the reversible transfer of adenosine triphosphate (ATP) to polyphosphate [17,18,19]. Some species regenerate phosphorus when the N:P ratio is below 25:1 [8]. Other bacterial families sequester phosphorus by modifying a filiform extension of the cell [20,21,22,23]. Another mechanism for bacterial survival under phosphorus-limiting conditions is to increase the formation of intracellular signaling molecules, resulting in the release of adhesin proteins from the cell walls to attach better to surfaces [24,25,26,27,28].

These bacterial adaptations to phosphorus-limiting conditions and related EPS production can compromise membrane cleaning strategies. It has been suggested that the stratification of bacterial populations in the inner biofilm layers developed a base biofilm attached to the membrane that is harder to remove [29,30]. Previously, it was demonstrated that reducing the phosphorus concentration in the water from 6 µg P·L^−1^ (P in RO feed water after dosage of phosphorus-based antiscalants) to 3 µg P·L^−1^ (P in seawater) enhanced the hydraulic cleanability of reverse osmosis membranes and feed spacers [31]. However, it is unknown which bacterial communities grow under phosphorus-limiting conditions and if the bacterial community in the biofilm that remains after membrane flushing (by increasing the crossflow velocity of reverse osmosis systems) is of a different composition.

This study analyzes the bacterial communities developed in biofilms grown in membrane fouling simulators (MFSs) supplied with water at three dosed phosphorus concentrations (0, 3, and 6 µg P·L^−1^) and with a constant biodegradable carbon concentration of 125 μg C·L^−1^. After biofilm development, forward flushing was applied by increasing the crossflow velocity in the MFSs from 0.18 to 0.35 m·s^−1^ for one hour [32]. Membrane performance was monitored by analyzing the feed channel pressure drop increase. Biomass detachment was studied by performing membrane autopsies and quantifying ATP, total cell counts and EPS. To our knowledge, this was the first time a 16sRNA gene sequencing biomass analysis was formed in phosphorus-enriched and phosphorus-limited biofilms to analyze and compare the different bacterial communities present before and after forward flushing and its effect on biofilm development. Understanding the bacterial communities developed under phosphorus-limiting conditions can help to define better biofouling control protocols and membrane cleaning strategies for more sustainable desalination.

## 2. Materials and Methods

### 2.1. Experimental Setup and Operational Parameters during Biofilm Growth

Dechlorinated tap water supplemented with nutrients (Table 1), containing an ultra-low phosphorus concentration of ≤0.3 µg PO_4_-P·L^−1^ measured as orthophosphate according to Javier et al. (2020, 2021) [9,31,33], was used as the feed water for this study. The tap water was produced through seawater reverse osmosis (SWRO) desalination at the King Abdullah University of Science and Technology desalination plant (Thuwal, Jeddah, Saudi Arabia). Previous studies have shown that the tap water used for this experiment is suitable for biofilm studies [10,34,35,36]. The tap water elemental composition can be found in Table S1 of the Supplementary Material.

**Figure 1:**
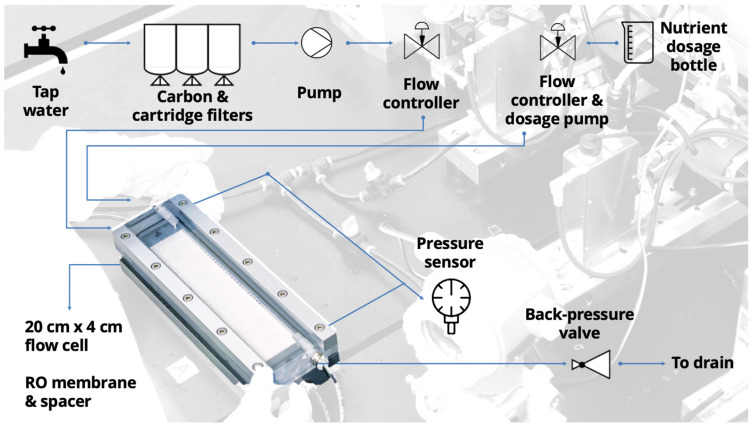
Lab-scale experimental setup (picture and schematic diagram).

The lab-scale experimental setup (Figure 1) consisted of (i) granular activated carbon (GAC) filter, (ii) two cartridge filters, (iii) a water pump, (iv) a flow controller, (v) a nutrient dosage pump, (vi) a membrane fouling simulator with inlet and outlet orifices for pressure drop measurements (MFS: [37]), (vii) a differential pressure sensor (Delta bar, PMD75, Endress+Hauser, Switzerland) to monitor the pressure drop over the feed channel, and (viii) a back pressure valve (Bronkhorst, Ruurlo, The Netherlands). The GAC (filter housing model: UPS BB3 (AWF-UPS-3H-20B); cartridge model: sediment-carbon (AC-SC-10-NL)) was used to remove the residual chlorine in the feed water. The two cartridge filters (pore size 4 µm) were placed after the GAC filter to remove any particles in the water that passed the GAC filter. The membrane and spacer placed in the MFS were a reverse osmosis (RO) polyamide membrane with the dimensions of 20 cm × 3.5 cm and a 34 mil (864 µm)-thick feed channel spacer, taken from a new commercially available spiral wound membrane element (TW30-4040, DOW FILMTEC, Miami, FL, USA). The hydraulics in the MFS, the membranes, and the spacers used in this study are representative of SWRO membrane systems [10].

Previous research showed that the first and strongest membrane performance parameter that affects biofilm development is the feed channel pressure drop [38]. Twelve fully independent membrane fouling simulators (Table 1) dosed with three different phosphorus concentrations, 0 μg P·L^−1^, 3 μg P·L^−1^, and 6 μg P·L^−1^, and with a dosed assimilable organic carbon concentration of 125 μg C·L^−1^ were run in parallel in a crossflow mode at a constant pressure of two bar without permeate production [37,39]. The membrane performance was monitored by analyzing the feed channel pressure drop increase over time. The average initial pressure drop registered in each MFS was 35 ± 5 mbar.

During biofilm growth, the feed water was pumped through the MFS at a flow rate of 17 L·h^−1^, equivalent to a linear flow velocity of 0.16 m·s^−1^, representing practical conditions at membrane filtration installations [40]. Analytical grade glucose, sodium nitrate, and sodium phosphate from Sigma Aldrich (Darmstadt, Germany) were added to the feed water to enhance biofilm growth in the MFSs. Sodium hydroxide was added to the nutrient solution to set the pH value at 11 to restrict bacterial growth. The high pH value of the nutrient solution did not affect the feed water pH of 7.8, as the nutrient solution was dosed at a low rate of 0.03 L·h^−1^, compared to the feed water flow of 17.0 L·h^−1^ [41]. The same dosed assimilable organic carbon and nitrogen concentrations of 125 µg C·L^−1^ and 25 µg N·L^−1^ were dosed to all MFSs, to only analyze the effect of varying phosphorus concentrations in the feed water on the bacterial communities developed. The 125 µg C·L^−1^ assimilable organic carbon concentration of glucose was used based on measurements performed at a desalination plant [42]. The dosed phosphorus concentrations for this study, 3 µg P·L^−1^ and 6 µg P·L^−1^, were selected based on phosphorus measurements that are typically present in seawater and the RO feed water after the addition of phosphorus-based antiscalants [31,43]. The selected dosed phosphorus concentration of 0 µg P·L^−1^ was used as removing the phosphorus concentration from the feed water has been suggested as a biofilm control strategy [12,44].

### 2.2. Forward Flushing and End of the Experiment

After biofilm development, forward flushing was initiated to duplicate the MFSs for each phosphorus-dosed condition by increasing the crossflow velocity in the MFSs from 0.18 to 0.35 m·s^−1^ for one hour. Techniques that allow the optimization of processes without increasing costs are being considered in relation to desalination operations. Therefore, we selected forward flushing as an easy-to-implement and environmentally friendly strategy to clean the biofilm from the membrane without chemical dosage. Forward flushing was performed after 140% feed channel pressure drop increases for biofilms grown at 3 µg P·L^−1^ (after 20 days) and 6 µg P·L^−1^ (after 5 days), and after 18% feed channel pressure drop increases in MFS operation for biofilms grown at 0 µg P·L^−1^ (after 20 days). The pressure drop increase of 140% simulated the pressure drop increase of 15% over the lead RO element of the first stage pressure vessel. In practice, cleaning protocols are applied when the lead element increases its pressure drop to 15% [32]. We stopped the MFSs of biofilms grown at 0 µg P·L^−1^ at the same time as the MFSs of biofilms grown at 3 µg P·L^−1^ as there was not a significant increase in the feed channel pressure drop after 20 days of MFS operation. MFSs were immediately stopped and sampled for biofilm analysis before and after forward flushing.

### 2.3. Optical Coherence Tomography

Biofilms in the MFSs were visualized in-situ on day 0 and at the end of the experiment using a spectral-domain Optical Coherence Tomography system (Thorlabs Ganymede OCT System, Lübeck, Germany), equipped with a central light source wavelength of 930 nm. The OCT 5× telecentric scan lens (Thorlabs LSM03BB, Lübeck, Germany) provides a maximum scan area of 100 mm^2^. OCT captures the intensity signal of the scattered media in two dimensions (XZ) by using coherent light. OCT can provide three-dimensional (3D) representations (XYZ) in seconds by combining two-dimensional images. For visualization purposes, six three-dimensional images were taken at each MFS’ inlet, middle, and outlet position. Each 3D image consisted of 278 two-dimensional images. Images were taken at a high-resolution frequency of 36 kHz, with a refractive index of 1.33. The images had a length of 5.00 mm and a depth of 1.00 mm with a pixel size in the x-direction of 18.00 μm and in the z-direction of 2.13 μm. The images shown in this study were representative of all the images analyzed.

### 2.4. Biomass Quantification

#### 2.4.1. Adenosine Triphosphate

MFSs were stopped and disassembled for biomass quantification at the end of the experiments. We retrieved membrane and feed spacer coupons of 4 × 4 cm^2^ from the MFS inlet and outlet positions to analyze the accumulated adenosine triphosphate (ATP). The coupons were then placed in a capped tube containing 40 mL of sterile tap water for ATP analysis. Next, the tubes were vortexed (for a few seconds) and placed in an ultrasonic water bath (Branson, 5510MTH, California, USA, output 135 W, 40 kHz) to detach the biomass from the membranes and spacers until the liquid was homogenous. The water collected from the tubes was used as a sample to determine ATP concentrations in the biofilms. Samples were measured in duplicates. We used a luminometer (Celsis Advance, Charles River Laboratories, Inc., Wilmington, MA, USA) for ATP measurements.

#### 2.4.2. Total Cell Count

We followed the protocol reported by Neu et al. (2019) [45] to quantify the total bacterial cell counts (TCC) in the biofilm using flow cytometry. We took coupons of 4 × 2 cm^2^ of the biofouled membrane and spacer from the MFS’ inlet and outlet positions. The coupons were then placed in a capped tube with 40 mL ultrapure water. The samples were vortexed and sonicated for 2 min to detach the biomass from the membrane and spacer. A sample of 700 µL was taken from the tube and stained with 7 µL·mL^−1^ SYBR Green I (100×) diluted from a 10,000× stock solution (Molecular Probes, Eugene, OR, USA). Next, the samples were incubated in the dark at 35 °C for 10 min. A BD Accuri C6 flow cytometer (BD Accuri Cytometers, Brussels, Belgium) equipped with a 50 mW laser with a fixed emission wavelength of 488 nm was used for TCC measurements. Fluorescence intensity was collected at FL1 = 533 ± 30 nm, FL3 > 670 nm, with sideward- and forward-scattered light intensities also being obtained. All data were processed with the BD Accuri CFlow^®^ software [46,47]. Electronic gating was used to select SYBR green-labeled signals to quantify the total bacterial cell count following the procedure described by Hammes and Egli, (2005) [48].

#### 2.4.3. Extraction and Quantification of Extracellular Polymeric Substances (EPS)

Extracellular polymeric substances were quantified by extracting 4 × 4 cm^2^ coupons of the biofouled membrane and spacer from the MFS. The coupons were placed into tubes containing 10 mL of phosphate-buffered saline solution (PBS). The tubes were vortexed for two minutes and sonicated for five minutes to separate the biomass from the membranes and feed spacers. We extracted the EPS following the formaldehyde–NaOH method established by (Liu and Fang, 2002) [49]. In brief, the water collected from the tubes was used as a sample for EPS extraction. A solution of 0.06 mL formaldehyde (36.5%; Sigma-Aldrich, St. Louis, MO, USA) was added to the samples and incubated at 4 °C for 1 h. Next, 4 mL 1 N NaOH was added to the samples and incubated at 4 °C for 3 h, then centrifugated for 20 min at 20,000× *g*. The supernatant was filtered through a 0.2 µm pore-sized membrane and dialyzed using a 3500 Da dialysis membrane (Thermo Fisher Scientific, Waltham, MA, USA) for 24 h. Samples were then lyophilized for 48 h and resuspended in 10 mL of ultrapure water. We quantified the biofilm’s carbohydrate concentration following the sulfuric acid phenol method [50]. In brief, 200 µL of the sample was mixed with 600 µL sulfuric acid and 120 µL 5% phenol. The samples were then incubated at 90 °C for 5 min and left to cool down. The carbohydrate absorbance at 490 nm was measured using a Spectra A max 340pc microplate reader (Molecular Devices, San Jose, CA, USA). We quantified the protein concentrations using bovine serum albumin (BSA) as a standard, using a BCA protein assay kit (Thermo Scientific Inc., Portsmouth, NH, USA) according to the manufacturer’s guidelines. The protein absorbance at 562 nm was measured using a Spectra A max 340pc microplate reader.

We calculated the bacterial and EPS relative abundance by multiplying the total cell count per cm^2^ by the average dry weight (between 83 and 1172 fg) of single bacterial cells reported in the literature [51]. The dry weight per cm^2^ was added to the total EPS per cm^2^ to obtain the total biofilm per cm^2^. The relative proportion of bacterial cells and EPS was then calculated.

### 2.5. DNA Extraction and Illumina Sequencing

Coupons of 4 × 4 cm^2^ of the biofouled membrane and spacer were used to extract microbial genomic DNA using the DNeasy^®^ PowerWater^®^ kit purchased from Qiagen (Maryland, USA) as per the manufacturer’s protocol. The concentration of extracted DNA was confirmed using the Qubit^™^ dsDNA BR assay kit with the Qubit^®^ 2.0 Fluorometer (Thermo Fisher Scientific, USA). Afterward, the extracted microbial community DNA was processed, and sequencing libraries were prepared for DNA sequencing in DNASense’s laboratory (Aalbirg, Denmark) by performing 16S rRNA gene-based high-throughput sequencing on the Illumina MiSeq platform. The forward (515F: GTGYCAGCMGCCGCGGTAA) and reverse (806R: GGACTACNVGGGTWTCTAAT) primers were designed to amplify the V4 region of the 16S rRNA gene [52,53]. The taxonomy of 16S rRNA sequences was assigned using the Ribosomal Database Project (RDP) classifier [54] based on the SILVA 16S rRNA database (SSU123). The raw sequencing data were processed using the research standard UPARSE workflow and analyzed through RStudio using the ampvis2 package developed at Aalborg University in Denmark. The abundances of the species presented in the analysis represent the count of each bacterial 16S rRNA gene in the sample. Bacterial community analysis was performed using the DNASense app https://dnasense.shinyapps.io/dnasense/ (access date: 1 September 2021). The bacterial alpha diversity was calculated using the Shannon–Weaver diversity index. The Shannon index increases as both species richness and evenness increase. The sequences were compared for their Bray–Curtis similarities and represented graphically for spatial distribution in a Principal Coordinates Analysis (PCoA) plot of 12 samples and 100 OTUs [55]. Before the analysis, OTU’s that were not present in more than 0.1% relative abundance in any sample were removed. The data were transformed initially by applying the Hellinger transformation [56]. The relative contributions (eigenvalues) of each axis to the total inertia in the data are indicated in percentages at the axis titles. Sequence reads for this study were submitted to the National Center for Biotechnology Information (NCBI).

## 3. Results

### 3.1. Feed Channel Pressure Drop Restoration and Visualization of Biofilm Removal

**Figure 2:**
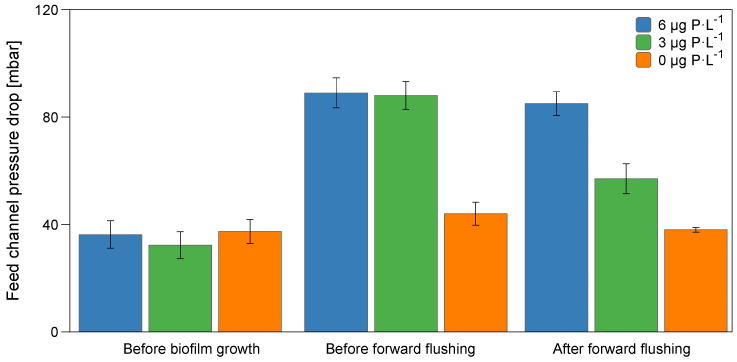
Feed channel pressure drop before biofilm growth, before forward flushing (crossflow velocity of 0.18 m·s^−1^), and after forward flushing for 1 h, for the biofilms grown at 0 μg P·L^−1^, 3 μg P·L^−1^, and 6 μg P·L^−1^ in the feed water with a dosed assimilable organic carbon concentration of 125 μg C·L^−1^. The average and standard deviation of independent duplicate experiments are shown.

This study analyzed the bacterial communities developed in biofilms grown in membrane fouling simulators (MFSs) supplied with water under three phosphorus conditions, 0 µg P·L^−1^ (simulating P removed from seawater), 3 µg P·L^−1^ (P in seawater), and 6 µg P·L^−1^ (P after the addition of phosphate-based antiscalants), with a constant biodegradable carbon concentration of 125 μg C·L^−1^. The membrane performance was monitored by analyzing the feed channel pressure drop increase over time. After biofilm development, forward flushing was initiated by increasing the crossflow velocity in MFSs from 0.18 to 0.35 m·s^−1^ for one hour. Forward flushing was selected as an easy-to-implement and environmentally friendly strategy to remove the biofilm from the membrane without chemical dosage. Figure 2 shows the feed channel pressure drop at the beginning of the experiment, before and after forward flushing. On day 0, all MFSs had an initial feed channel pressure drop of 35.3 ± 5 mbar. Before forward flushing, the pressure drops for biofilms grown at 0, 3, and 6 μg P·L^−1^ increased to 44.0 ± 4, 88.1 ± 5, and 89.0 ± 5 mbar, respectively. Forward flushing was initiated by increasing the crossflow velocity to 0.35 m·s^−1^ for 1 h, and the pressure drop decreased to 38.0 ± 4 mbar, 57.0 ± 5 mbar, and 85.0 ± 4 mbar for biofilms grown at 0, 3, and 6 μg P·L^−1^, respectively. A higher pressure drop percentage (60%) was recovered for biofilms grown at 3 μg P·L^−1^, compared to 0 and 6 μg P·L^−1^. In conclusion, increasing the phosphorus concentration to 6 μg P·L^−1^ in the feed water by adding phosphate-based antiscalants had a detrimental effect on the hydraulic cleanability and the MFS’ feed channel pressure drop restoration.

**Figure 3:**
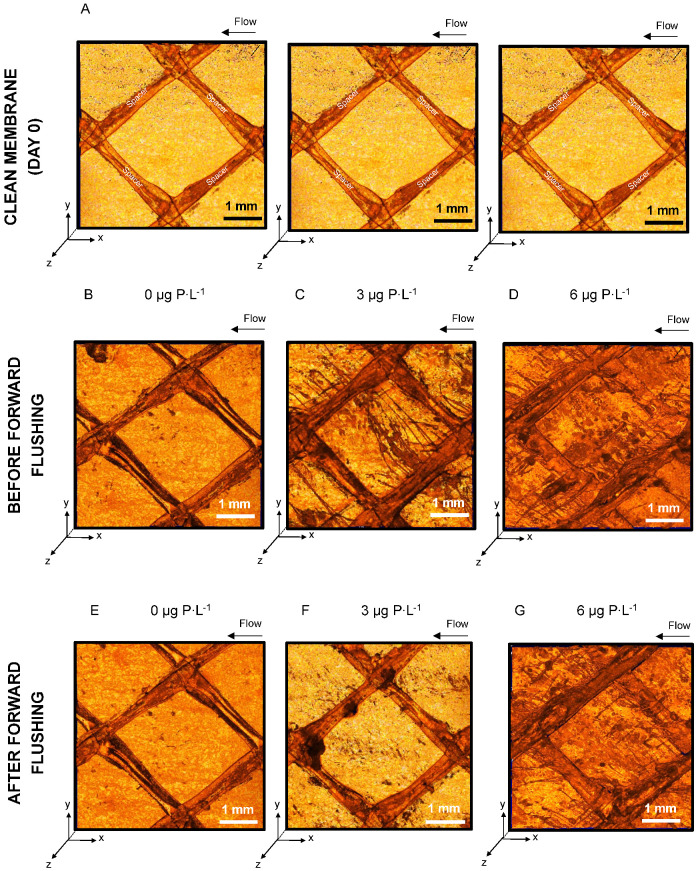
Three-dimensional visualization (top view) of (**A**) a clean membrane and spacer on day 0 before nutrient dosage imaged with OCT. The OCT images (5 mm length) show the biofilms grown under feed water conditions of (**B**,**E**) 0 µg P·L^−1^, (**C**,**F**) 3 µg P·L^−1^, and (**D**,**G**) 6 µg P·L^−1^ with a dosed assimilable organic carbon concentration of 125 μg C·L^−1^, before and after forward flushing by increasing the crossflow velocity from 0.18 to 0.35 m·s^−1^ for 1 h. Six three-dimensional images were taken at the inlet, middle, and outlet positions of each MFS. The arrow indicates the flow direction.

Figure 3 shows the top view of the three-dimensional OCT images at the beginning of the experiment, before and after forward flushing. OCT images confirmed the presence of biofilm on membranes and spacer for the 0 µg P·L^−1^ feed water conditions (Figure 3E). The images show a higher biofilm development for biofilms grown at 6 µg P·L^−1^, compared to 0 and 3 µg P·L^−1^ biofilms. Visually, a stronger biofilm removal was achieved at a 3 µg P·L^−1^ concentration of supplemented phosphorus in the feed water (Figure 3F), compared to the biofilm removal at 0 and 6 µg P·L^−1^ (Figure 3E,G). In conclusion, the system performance was better restored maintaining the phosphorus concentration of seawater (3 µg P·L^−1^), avoiding the dosage of phosphate-based antiscalants (6 µg P·L^−1^).

### 3.2. Biomass Characterization

**Figure 4:**
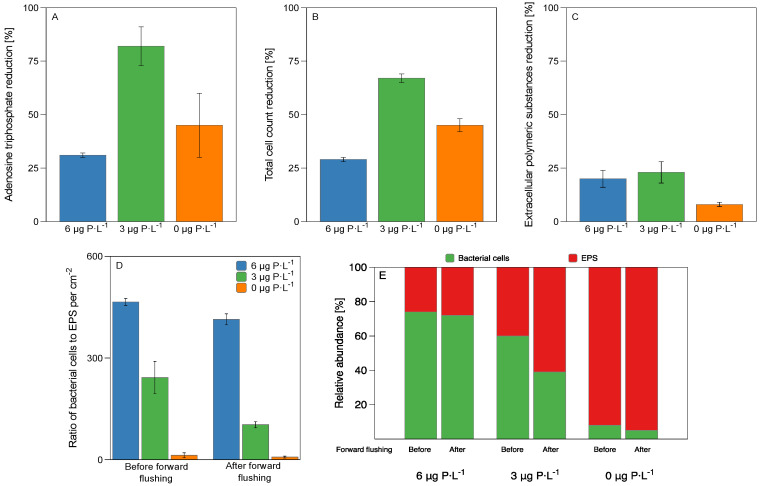
Biofilm characterization of the biofouled membrane and spacer before and after MFS forward flushing. (**A**) adenosine triphosphate reduction, (**B**) total cell count reduction, (**C**) extracellular polymeric substances (EPS) reduction, (**D**) ratio of bacterial cells to EPS per cm^2^, and (**E**) relative abundance of cells and EPS for the biofilms grown at 0 μg P·L^−1^, 3 μg P·L^−1^, and 6 μg P·L^−1^ feed water conditions with a dosed assimilable organic carbon concentration of 125 μg C·L^−1^, before forward flushing (crossflow velocity of 0.18 m·s^−1^) and after MFS forward flushing by increasing the crossflow velocity to 0.35 m·s^−1^ for 1 h. The average and standard deviation of independent duplicate experiments are shown.

The biomass present on the membrane and spacer was characterized before and after forward flushing. Table 2 shows the data for the adenosine triphosphate (ATP), total cell count (TCC), proteins, and extracellular polymeric substances (EPS) in terms of proteins and carbohydrates for the biofilms grown at the three different dosed phosphorus concentrations. Figure 4A shows that there was a higher ATP reduction, 82 ± 9%, for biofilms grown at 3 µg P·L^−1^ compared to biofilms grown under 0 and 6 µg P·L^−1^, where ATP removal was 45 ± 15% and 31 ± 1%, respectively. Figure 4B shows that the TCC removal after forward flushing was also higher, 67 ± 2%, for biofilms grown at 3 µg P·L^−1^ compared to biofilms grown at 0 and 6 µg P·L^−1^, where TCC reduction was 45 ± 3% and 29 ± 1%, respectively. Forward flushing removed similar EPS quantities for biofilms grown at 3 and 6 µg P·L^−1^, 23 ± 5% and 20 ± 4%, respectively, compared to biofilms grown at 0 µg P·L^−1^, where the EPS reduction was 8 ± 1% (Figure 4C). In summary, a stronger removal in terms of ATP, TCC, and EPS was achieved by forward flushing for biofilms grown at 3 μg P·L^−1^ compared to 0 and 6 µg P·L^−1^ biofilms. Figure 4D shows that the ratio of bacterial cells to EPS per cm^2^ decreased as the phosphorus concentration decreased, with values of 465 ± 90, 242 ± 107, and 13 ± 7 for the biofilms grown at 6, 3, and 0 μg P·L^−1^, respectively. After forward flushing, the ratio decreased to 413 ± 16, 103 ± 9, and 7 ± 3 for the biofilms grown at 6, 3, and 0 μg P·L^−1^, respectively. The relative abundance of bacteria cells and EPS was calculated based on the average dry weight (between 83 and 1172 fg) of single bacterial cells reported in the literature [51]. Figure 4E shows that the proportion of bacterial cells decreased on biofilms analyzed before forward flushing, and the EPS concentration increased as the phosphorus concentration decreased in the feed water. In summary, at lower phosphorus concentrations, there was less ATP and TCC but more EPS production per cell.

### 3.3. Bacterial Diversity and Community Analysis

**Figure 5:**
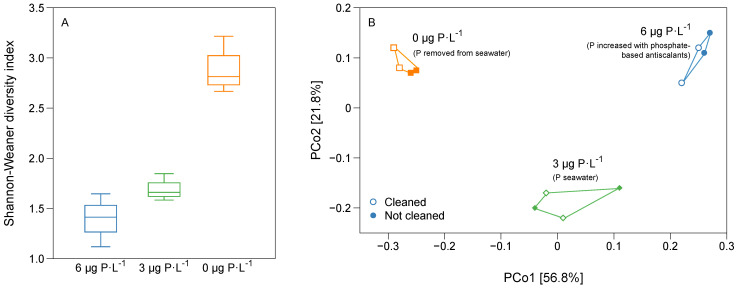
α and β bacterial diversity for biofilms grown under the three phosphorus concentration conditions. (**A**) Shannon-Weaner diversity index (α diversity) and (**B**) principal coordinates analysis (β diversity) based on the Bray-Curtis distance metric. Each point represents the microbial community in a specific sample for the biofilms grown under 0 µg P·L^−1^, 3 µg P·L^−1^, and 6 µg P·L^−1^ feed water conditions with a dosed assimilable organic carbon concentration of 125 μg C·L^−1^. Empty points show the cleaned MFSs obtained by increasing the crossflow velocity from 0.18 to 0.35 m·s^−1^ for 1 h. The filled points represent the MFSs that were not cleaned. Distance between the sample dots signifies similarity; the closer the samples are, the more similar their microbial compositions.

Figure 5A shows the alpha bacterial diversity calculated by the Shannon–Weaner diversity index. The Shannon–Weaner diversity index increased (1.41, 1.66, and 2.81) as the phosphorus concentration in the feed water decreased from 6, 3, and 0 μg P·L^−1^, respectively, suggesting a more diverse community on biofilms grown under low phosphorus concentration conditions. The principal coordinates analysis shows the reproducibility of the data (Figure 5B). Four independent MFSs were run, and the bacterial community results show nearby clustering along the PCo1 and PCo2 axes for each phosphorus concentration condition. The principal coordinates analysis shows that different bacterial communities formed when varying the phosphorus concentration in the feed water. The bacterial communities did not change after the forward flushing of MFSs, suggesting a homogenous distribution of bacterial communities across the biofilm height. To summarize, a more diverse bacterial community developed as the phosphorus concentration decreased, and the bacterial diversity did not significantly change after forward flushing.

**Figure 6:**
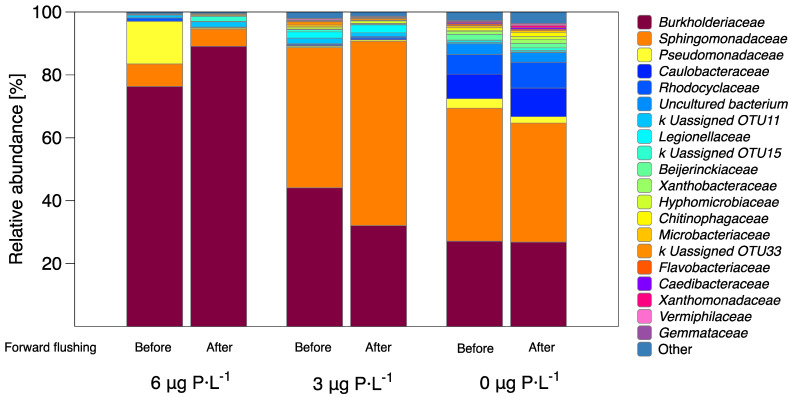
Taxonomic distributions of bacterial community at the family level for the biofilms grown under a dosed phosphorus concentration of 0 µg P·L^−1^, 3 µg P·L^−1^, and 6 µg P·L^−1^ with a dosed assimilable organic carbon concentration of 125 μg C·L^−1^ in the feed water, before forward flushing (crossflow velocity of 0.18 m·s^−1^) and after forward flushing by increasing the crossflow velocity to 0.35 m·s^−1^ for 1 h.

*Burkholderiaceae* and *Sphingomonadaceae* were the two prominent bacterial families that dominated the bacterial community during biofilm growth for the three phosphorus-dosed conditions (Figure 6). For biofilms grown at 6 µg P·L^−1^, the *Burkholderiaceae* family represents 76.3% of the total bacterial community, followed by *Pseudomonadaceae* with 13.5%, and *Sphingomonadaceae* with 7.2%. Table 2 shows a higher protein concentration for the biofilms grown at 6 µg P·L^−1^ condition compared to biofilms grown at 0 and 3 µg P·L^−1^. *Burkholderiaceae* has been associated with a high adhesin protein production in the cell wall [24,57]. In contrast, a higher relative abundance of the *Sphingomonadaceae* family was present for biofilms grown at 3 and 0 µg P·L^−1^ conditions, with values of 44.6% and 42.3%, respectively, compared to biofilms grown at 6 µg P·L^−1^. *Sphingomonadaceae* are related to the production of extracellular polymeric substances [58,59,60,61]. *Caulobacteraceae* represented 7.8% of the relative bacterial abundance for biofilms grown at 0 µg P·L^−1^, while it had lower abundance for biofilms grown at 3 and 6 µg P·L^−1^. The most predominant bacterial families that stayed after the forward flushing of MFSs for the three phosphorus conditions were *Burkholderiaceae* and *Sphingomonadaceae* (Table 3). In conclusion, the bacterial biofilm composition remained the same before and after forward flushing, indicating a homogenous bacterial composition over the biofilm height.

## 4. Discussion

### 4.1. Bacterial Adaptation to Phosphorus-Limiting Conditions

Understanding differences in the biofilm developed in membrane systems under varying nutrient compositions in the feed water is vital to optimize membrane cleaning strategies. Previous studies have shown that minute changes in the phosphorus concentration in the feed water impact bacterial planktonic cell growth and the overall development of biofilm [7,8,9]. Some of the bacterial adaptations to phosphorus-limiting conditions include, but are not limited to, (i) increased EPS production, (ii) phosphorus accumulation, regeneration, and sequestration, and (iii) the regulation of adhesin proteins at the bacterial cell wall. This study assessed the bacterial communities and biomass characteristics developed before and after forward flushing by increasing the crossflow velocity for 1 h, in biofilms grown in membrane fouling simulators (MFSs) supplied with water with three phosphorus conditions (0, 3, and 6 µg P·L^−1^) and with a constant biodegradable carbon concentration. 

#### 4.1.1. EPS Production

Vrouwenvelder et al. (2010) [12] and Kim et al. (2014) [13] showed that fewer individual active cells developed under phosphorus-limiting conditions. Recent research focused on quantifying the EPS of phosphorus-limited biofilms; Desmond et al. (2018) [16] and Javier et al. (2020) [9] showed that EPS production increased under phosphorus-limiting conditions. Figure 4E shows that as the phosphorus concentration decreased, the ratio of EPS production per bacteria cell increased, most probably to protect bacteria under stressed conditions and maximize nutrient intake [16]. The relative abundance of the bacterial family *Sphingomonadaceae* started increasing at 3 µg P·L^−1^. *Sphingomonadaceae* are related to the production of extracellular polymeric substances [58,59,60,61]. Previously it was demonstrated that when lowering the phosphorus concentration in the feed water from 6 µg to 3 µg P·L^−1^, an enhanced detachment was observed, explained by less protein production and more soluble polymers being present in the EPS matrix [31]. In this study, a better biofilm removal (in terms of ATP, TCC, and EPS) was achieved after forward flushing for biofilms grown at 3 µg P·L^−1^ compared to the 0 and 6 µg P·L^−1^ conditions (Figure 2). The enhanced biofilm removal can be explained in terms of the biofilm localization. For the 0 µg P·L^−1^ condition, the biofilm spread evenly on the membrane (Figure 3), not causing a significant increase in the feed channel pressure drop (Figure 2). Therefore, the biofilm showed less impact on changes in the crossflow velocity. It was suggested that the type of EPS matrix influences the mechanical biofilm properties and biofilm localization in the flow channel [65]. The biofilm composition for the three dosed phosphorus was different in terms of the relative abundance of cells and EPS. At a higher dosed phosphorus concentration of 6 µg P·L^−1^, the biofilm composition was approximately 80% cells and 20% EPS. At the lowest dosed phosphorus concentration of 0 µg P·L^−1^, the biofilm composition was the opposite, showing 5% cells and 95% EPS. It was interesting to see that the biofilm that was better removed (3 µg P·L^−1^) had a balanced ratio of cells to EPS, around 60% cells and 40% EPS, compared to the biofilms grown at 0 and 6 µg P·L^−1^. In summary, we observed a trend in which the ratio of cells to EPS could influence the biofilm localization, the mechanical biofilm properties, and the response to shear forces. 

#### 4.1.2. Phosphorus Accumulation, Regeneration, and Sequestration

Some microorganisms, under phosphorus-limiting conditions, respond by accumulating phosphorus within their cells. The bacterial family *Burkholderiaceae* has been identified as a polyphosphate-accumulating organism (PAO). An enzyme catalyzes the microbial synthesis of intracellular polyP through the reversible transfer of ATP to polyP (Mullan et al., 2002; Song et al., 2008; Zeng et al., 2017). In this study, the bacterial family *Burkholderiaceae* was present for the three dosed phosphorus concentrations, being more abundant on biofilms grown at 6 µg P·L^−1^. The relative abundance for the *Burkholderiaceae* family did not change after forward flushing. Other species, aside from accumulating, can regenerate phosphorus. The bacterial family *Pseudomonadaceae* was reported to regenerate phosphorus when the N:P ratio is below 25:1 [8]. This bacterial family was the second most present in biofilms grown at 6 µg P·L^−1^ when the N:P ratio was the lowest, at 4.2:1, compared to the other phosphorus-dosed conditions (Figure 6). Similarly, different species modify their morphology to sequester phosphorus to adapt to nutrient limitation. Most species of the family *Caulobacteraceae* produce a filiform extension of the cell, known as prosthecum or stalk. Phosphorus-starved cells produce stalks as much as 30 times longer compared to cells growing in phosphorus-enriched conditions. The increase in surface/volume ratio caused by stalk elongation during phosphorus limitation allows the cell to take up phosphorus more efficiently, and therefore, act as a phosphorus scavenger [20,21,22,23]. In this study, the bacterial family *Caulobacteraceae* was the third most predominant species in biofilms grown at the dosed 0 µg P·L^−1^ condition, proving that even under ultra-trace reactive phosphorus concentration in the feed water (≤0.3 PO_4_-P·L^−1^), some bacterial families start to predominate to provide enough nutrients to the biofilm matrix.

#### 4.1.3. Adhesin Protein Production

**Figure 7:**
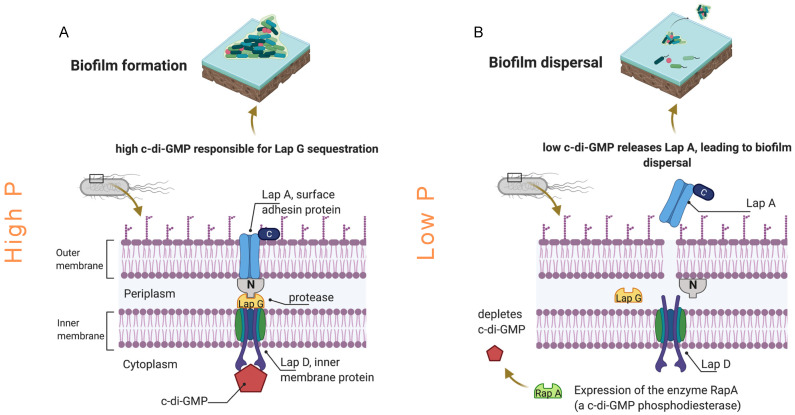
Model for biofilm formation and dispersal on the membrane at high and low phosphorus concentrations. (**A**) At high phosphorus concentration, there is an increase in the production of the intracellular signaling molecule cyclic dimeric guanosine monophosphate (c-di-GMP), which binds the inner membrane protein Lap D. LapD interacts with the protease LapG in the periplasm. LapG cleaves the N-terminal of LapA, an outer membrane adhesin protein. LapA is associated with surface attachment, and therefore, biofilm formation. (**B**) Phosphorus limitation (low P) promotes the expression of the enzyme RapA, which depletes the production of the secondary messenger c-di-GMP. The LapD/Lap G complex dissociates, and the LapA protein is released from the cell surface, promoting biofilm dispersal. Adapted from [25,26,28].

Some bacteria respond differently under phosphorus-limiting and phosphorus-enriched conditions by developing mechanisms to attach more strongly to surfaces. Figure 7 shows a graphical explanation of the mechanisms proposed in the literature for protein production and biofilm formation under phosphorus-enriched and phosphorus-starved conditions. As the phosphorus concentration increases, some bacterial families start producing more intracellular signaling molecules that increase the outer membrane adhesin proteins, associated with surface attachment [24,25,26,27,28]. On the contrary, some of these adhesin proteins are released from the cell surface when phosphorus is depleted, promoting biofilm dispersal. It has been proven that some species of the bacterial family *Burkholderiaceae* increase the protein production at bacteria cell walls, which enables the bacterial cells to attach better to the surfaces [57]. In this study, the relative abundance of the bacterial family *Burkhoderiacea* was higher for biofilms grown at 6 µg P·L^−1^ compared to 0 and 3 µg P·L^−1^ (Figure 6). The lower protein production can be a possible explanation as to why the biofilm grown at 3 µg P·L^−1^ had an enhanced hydraulic cleanability after forward flushing compared to the other phosphorus-dosed conditions (Figure 2).

### 4.2. Homogenous Bacterial Community Composition throughout the Biofilm Height

Our study involved biofilm analysis in reverse osmosis MFSs. However, different studies in wastewater have proven that biofilms stratify into aerobic, anoxic, and anaerobic zones to achieve the removal of organic pollutants [29,30,66,67,68,69]. If biofilms stratify across the biofilm height in reverse osmosis membrane systems, then the base biofilm layer would be harder to remove, compromising membrane cleaning strategies. Desmond et al. (2018) [70] proved that in nutrient-enriched and river water biofilms, a stratification in cohesion was observed over the biofilm’s depth. A cohesive base layer remained attached to the membrane surface when increasing the shear conditions for these nutrient-enriched biofilms. Conversely, phosphorus-limited biofilms showed uniform structural properties over the biofilm’s depth. Under high shear forces, the phosphorus-limited biofilms detached by sloughing from the membrane surface, indicating more susceptible detachment. Our results agree with previous research where an enhanced hydraulic cleanability was observed for biofilms grown under phosphorus-limiting conditions [31,70]. Moreover, the bacterial families remained the same after forward flushing in this study, suggesting a homogenous bacterial population across the biofilm height (Figure 6). Therefore, non-expensive physical cleaning methods of membranes can be applied to enhance the membrane system performance.

### 4.3. Practical Implications and Future Research

The water cost from desalination sources has dramatically decreased since 1980, reaching a plateau of around 1 USD per m^3^ from year 2000. However, seawater reverse osmosis desalination is still considered an energy-intensive technology [71]. The main costs of fouling are associated with early membrane replacement and energy costs [72]. Therefore, research is moving into greener desalination to reduce the overall water cost. In terms of biofouling control, some strategies include, but are not limited to, (i) altering the nutrient conditions, (ii) modifying the morphology of membranes and spaces, and (iii) reducing the chemicals and the toxicity of the chemicals dosed during the desalination process [10,73,74,75]. This study demonstrated that even a minor variation in the dosed phosphorus concentration in the feed water alters the bacterial community and the EPS produced within the biofilm, resulting in a different effect on the membrane performance decline and the hydraulic membrane cleanability. An easy-to-implement strategy to improve the overall system performance is to stop the addition of phosphate-based antiscalants. The increase in phosphorus concentration in the feed water develops biofilms that are more attached to membranes and are harder to be removed by hydraulic cleaning methods. Membrane cleaning strategies should consider the feed water’s C:N:P ratio to increase their effectiveness. Future research should focus on manipulating the feed water nutrient concentrations with antiscalants used in practice under permeation conditions to define a biofilm with known characteristics. These biofilms could be easier to control and remove without chemicals. Other cleaning strategies should be evaluated, such as reverse flushing, air backflushing, air bubbling, and CO_2_ addition [76,77,78,79], shifting the conventional chemical cleaning methods to a more sustainable approach for greener desalination [80].

## 5. Conclusions

This study analyzed the bacterial communities developed in biofilms grown in membrane fouling simulators (MFSs) supplied with water with three dosed phosphorus conditions (0, 3, and 6 µg P·L^−1^) and with a constant biodegradable carbon concentration of 125 μg C·L^−1^. The conclusions of the present study can be summarized as follows:

(i) A better biofilm removal (in terms of ATP, TCC, and EPS) was achieved by forward flushing for biofilms grown at 3 µg P·L^−1^ compared to the 6 µg P·L^−1^ conditions (Figure 4), explained by the biofilm localization (Figure 3) and the production of a balanced proportion of cells to EPS (Figure 4).
(ii) For biofilms grown at lower phosphorus concentration conditions, the ratio of EPS production per bacterial cell was higher than for higher phosphorus concentrations (Figure 4).
(iii) The relative abundance of main bacterial communities changed at varying dosed phosphorus concentrations in the feed water (Figure 6).
(iv) For the three dosed phosphorus concentration conditions in the feed water, the bacterial biofilm population remained the same after forward flushing, suggesting a homogenous bacterial community composition along the biofilm height (Figure 5 and Figure 6).

## Figures and Tables

**Table 1:** Experimental conditions for the study (all experiments were run in duplicates).

Dosed Carbon Concentration (µg C·L^−1^) as Glucose	Dosed Nitrogen Concentration (µg N·L^−1^) as Sodium Nitrate	Dosed Phosphorus Concentration (µg P·L^−1^) as Sodium Phosphate	C:N:P Ratio	Forward Flushing
125	25	0	100:20:0.24 *	No
		0	100:20:0.24 *	Yes
		3	100:20:2.4 *	No
		3	100:20:2.4 *	Yes
		6	100:20:4.8 *	No
		6	100:20:4.8 *	Yes

* The water phosphorus concentration was ≤0.3 µg PO_4_-P·L^−1^ measured as orthophosphate.

**Table 2:** Biomass parameters of biofilms grown at different phosphorus concentrations (all experiments were run in duplicates).

Study	Dosed Phosphorus Concentration	6 µg P·L^−1^	3 µg P·L^−1^	0 µg P·L^−1^
Adenosine triphosphate (ng·cm^−2^)	Before forward flushing	88.52 ± 6.55	24.54 ± 5.04	2.44 ± 0.41
	After forward flushing	61.07 ± 2.75	4.48 ± 0.49	1.34 ± 0.45
Total cell count (×10^7^ cells cm^−2^)	Before forward flushing	8.01 ± 0.28	2.96 ± 0.17	0.11 ± 0.01
	After forward flushing	5.70 ± 0.20	0.97 ± 0.02	0.06 ± 0.00
Proteins (µg·cm^−2^)	Before forward flushing	8.22 ± 1.05	7.21 ± 0.58	3.54 ± 0.12
	After forward flushing	7.77 ± 0.89	5.39 ± 1.16	3.50 ± 0.03
Extracellular polymeric substances in terms of proteins and carbohydrates (µg·cm^−2^)	Before forward flushing	17.22 ± 3.05	12.21 ± 1.58	8.39 ± 0.37
	After forward flushing	13.77 ± 1.89	9.39 ± 2.16	7.72 ± 1.40

**Table 3:** Summary of predominant bacterial families at different phosphorus concentrations.

This Experiment		Literature	
Dosed Phosphorus Concentration	Bacterial Family and Class Ordered from Higher to Lower Percentage of Relative Abundance	Effect on Biofilm Development Under Phosphate-Limiting Conditions	Reference
6 µg P·L^−1^	*Burkholderiaceae* (betaprotobacteria)	Adhesin protein production and P accumulation	[17,24,57]
	*Pseudomonadaceae* (gammaprotobacteria)	Increase in quorum-sensing signals that promote biofilm formation, P regeneration	[8,62,63,64]
	*Sphingomonadaceae* (alphaproteobacteria)	EPS production	[58,59,60,61]
3 µg P·L^−1^	*Sphingomonadaceae* (alphaproteobacteria)	EPS production	[58,59,60,61]
	*Burkholderiaceae* (betaprotobacteria)	Adhesin protein production and P accumulation	[17,24,57]
0 µg P·L^−1^	*Sphingomonadaceae* (alphaproteobacteria)	EPS production	[58,59,60,61]
	*Burkholderiaceae* (betaprotobacteria)	Adhesin protein production and P accumulation	[17,24,57]
	*Caulobacteraceae* (alphaproteobacteria)	Phosphorus sequestration	[20,21,22,23]

## Data Availability

Not applicable.

## Supplementary Materials

The following are available online at https://www.mdpi.com/article/10.3390/membranes11120928/s1, Table S1: Elemental analysis of Tap water.

## Abbreviations

ATP	Adenosine triphosphate
BSA	Bovine serum albumin
c-di-GMP	Cyclic dimeric guanosine monophosphate
EPS	Extracellular polymeric substances
MFS	Membrane fouling simulator
NCBI	National Center for Biotechnology Information
OCT	Optical Coherence Tomography
PAO	Polyphosphate-accumulating organisms
PBS	Phosphate-buffered saline solution
PCoA	Principal Coordinates Analysis
RDP	Ribosomal Database Project
RO	Reverse osmosis
SWRO	Seawater reverse osmosis
TCC	Total cell count
