# Supplementary material for: Phosphorus Concentration in Water Affects the Biofilm Community and the Produced Amount of Extracellular Polymeric Substances in Reverse Osmosis Membrane Systems
Source: Membranes (Basel). 2021 Nov 26;11(12):928. doi: 10.3390/membranes11120928 (PMC8707166; doi:10.3390/membranes11120928)
Supplement: Supplementary file 1 [file membranes-11-00928-s001.zip › membranes-1441615-supplementary.pdf]

membranes

Article

Phosphorus Concentration in Water Affects the Biofilm Community and the Produced Amount of Extracellular Polymeric Substances in Reverse Osmosis Membrane Systems

Luisa Javier [1], Laura Pulido-Beltran [1], Joop Kruithof [2], Johannes S. Vrouwenvelder [1,3] and Nadia M. Farhat [1,*]

[1] Water Desalination and Reuse Center (WDRC), Division of Biological and Environmental Science and Engineering (BESE), King Abdullah University of Science and Technology (KAUST), Thuwal 23955-6900, Saudi Arabia; luisa.javier@kaust.edu.sa (L.J.); laura.pulidobeltran@kaust.edu.sa (L.M.P.B.); johannes.vrouwenvelder@kaust.edu.sa (J.S.V.)
[2] Wetsus, European Centre of Excellence for Sustainable Water Technology, Oostergoweg 9, 8911 MA Leeuwarden, The Netherlands; joop.kruithof@planet.nl
[3] Faculty of Applied Sciences, Department of Biotechnology, Delft University of Technology, Van der Maasweg 9, 2629 HZ Delft, The Netherlands
* Correspondence: nadia.farhat@kaust.edu.sa

Supplementary material

Table S1. Elemental analysis of Tap water.

Elements	Tap water µg/L
Sodium (Na)	28600
Calcium (Ca)	3600
Potassium (K)	1930
Magnesium (Mg)	858.6
Iron (Fe)	124.7
Zinc (Zn)	74.4
Aluminum (Al)	17.3
Strontium (Sr)	9.0
Nickel (Ni)	4.8
Lead (Pb)	2.5
Gold (Au)	1.7
Copper (Cu)	1.7
Palladium (Pd)	1.2
Lithium (Li)	1.1
Chromium (Cr)	<0.001
Cobalt (Co)	ND
Rubidium (Rb)	1.0
Silver (Ag)	0.7
Mercury (Hg)	0.5
Scandium (Sc)	0.5
Manganese (Mn)	0.5
Barium (Ba)	0.5
Vanadium (V)	0.3
Titanium (Ti)	0.2
Beryllium (Be)	0.2

Cadmium (Cd)	0.07

ND=Not determined.
